# Macroelement Content (Na, K, Ca, and Mg) in Microwave Foods

**DOI:** 10.3390/nu17101678

**Published:** 2025-05-15

**Authors:** Christian E. García, Soraya Paz-Montelongo, Arturo Hardisson, Carmen Rubio, Ángel J. Gutiérrez, Dailos González-Weller, Javier Darias-Rosales, Samuel Alejandro-Vega

**Affiliations:** 1 Area of Toxicology, University of La Laguna, Canary Islands, 38071 La Laguna, Tenerife, Spain; christiangarto@gmail.com (C.E.G.); atorre@ull.edu.es (A.H.); crubio@ull.edu.es (C.R.); ajguti@ull.edu.es (Á.J.G.); salejand@ull.edu.es (S.A.-V.); 2 Programa de Doctorado en Ciencias Médicas y Farmacéuticas, Desarrollo y Calidad de Vida, University of La Laguna, 38071 La Laguna, Tenerife, Spain; 3 Health Inspection and Laboratory Service, Canary Health Service, 38006 Santa Cruz de Tenerife, Tenerife, Spain; dgonzal@ull.edu.es

**Keywords:** microwave-ready meals, macroelements, dietary intake, ICP-OES

## Abstract

Background/Objectives: The consumption of microwave ready meals has increased significantly in recent years due to a noticeable reduction in the available time to spend cooking. However, one of the issues about this type of diet is its nutrient intake. The main objective of this study was to determine the content of macroelements (Na, K, Ca, and Mg) in samples of animal origin (omelette, chicken curry, meatballs, shredded meat, and prawns), vegetable origin (vegetable garnish, round rice, pesto pasta, cream of vegetable soup, and chickpeas with spinach), and mixed origin (pizza, lasagna, seafood paella, cannelloni, and spaghetti Bolognese). Methods: The macroelement content was determined by ICP-OES (Inductively Coupled Plasma—Optical Emission Spectrometry) in 288 samples of different microwave foods. Results: Likewise, the possible difference in the content of macroelements after the microwave heating process was studied, and significant differences in the Ca content were observed in the three analyzed food groups, indicating that there may have been migration from the container to the food. The concentrations of Na and Ca in the tails of garlic prawns (9381 ± 3102 mg Na/kg fw and 845 ± 134 mg Ca/kg fw) stood out. The vegetable side dish stood out for its higher concentration of K (3424 ± 1319 mg/kg fw). Pizza registered the highest concentrations of the four macrolements within the group of foods of mixed origin. The study of the dietary intake indicated that the consumption of some animal-based products offered a contribution to the safe and adequate intake of Na of almost 50%, which could pose a risk of dietary overdose as Na is an element found in many foods. Conclusions: It is recommended to moderate the consumption of some of the dishes analyzed mainly because of the risk of the high intake of Na.

## 1. Introduction

Microwave ready meals are fifth-range foods. Ready meals entail the mixing and seasoning of animal and vegetable foods, with or without the addition of other authorized substances, contained in appropriate containers that are hermetically sealed and treated by heat or by another process to ensure their preservation, which are ready to be consumed after a simple treatment. The following are included in this definition: ready-to-eat cooked dishes, cooked dishes, and ready-to-eat dishes [1,2].

Microwaves are low-energy electromagnetic radiation that do not ionize food. They are applied to foods that contain water, causing vibrations in the molecules, generating heat and friction of these. Microwaves do not generate toxic substances or secondary metabolites that affect the palatability of food. However, it has been shown that they can reduce the content of some nutrients, such as thermolabile vitamins [3,4]. The migration process of chemical pollutants harmful to health [5] that are transferred from packaging and plastic bags to food has also been studied.

From recent studies, it is estimated that the consumption of microwaved meals has increased by 20% in the last five years [6]. According to Eating Better’s 2020 Instant Ready Meals Survey, 88% of adults in the UK eat prepared breakfasts and dinners or ready-to-cook foods, and two out of five people eat packaged meals each week [6].

The analysis of the consumption of ready meals is carried out through the latest report on food consumption in Spain for 2022 prepared by the Ministry of Agriculture, Fisheries and Food, which explains that compared with 2019, the evolution of ready meals is growing, that is, they are gaining relevance in Spanish households with an increase in volume of 11.0%. On average, ready meals represent 4.63% of the average budget that Spanish households allocate to food and beverage purchases, those being 2.62% of the total volume [7].

Ready-made soups and creams are the most consumed by the Spanish population with an intake per person of 6.03 kg per year, although their consumption decreased by 1.7% at the end of 2022, the equivalent of 0.11 kg less than during the previous period [7].

Frozen ready meals are the next most consumed within this category, with an average intake of 2.53 kg per person and study period. This amount also decreases compared with that of the previous year, 2021, resulting in a lower intake of 0.16 kg per person per year. Pizza dishes also have a significant amount ingested per person per year, specifically 2.28 kg. The drop in per capita consumption is very abrupt since the previous year per capita consumption was 2.47 kg/person/year, so around 0.19 kg per person are no longer consumed, the equivalent of 7.8% of the previous registered data [7].

Macroelements are minerals needed by the human body in amounts greater than 100 mg per day [8]. They are essential for various bodily functions, including maintaining bone health, electrolyte balance, and metabolism. Macroelements include calcium, potassium, sodium, and magnesium, among others [9].

Sodium (Na) and potassium (K) are essential for maintaining osmotic balance and nerve transmission, regulating ion exchange across cell membranes. Magnesium (Mg) is key to muscle function, protein synthesis, and enzyme activation. Calcium (Ca) is critical for bone and tooth formation, blood clotting, and muscle contraction. These macroelements are crucial for various vital functions in the body [10].

The objectives of this study were (1) to determine the content of macroelements (Na, K, Ca, and Mg) in microwave ready meals of animal, vegetable, and mixed origin by ICP-OES (inductively coupled plasma optical emission spectrometry); (2) to study the possible influence of microwave preparation on the content of macroelements; and (3) to evaluate the dietary intake of macroelements considering the reference values.

## 2. Material and Methods

### 2.1. Samples

A total of 150 samples of meals prepared for microwave preparation prior to cooking were analyzed, differentiating between animal (50), vegetable (50), and mixed (50) origins. The samples were purchased in supermarkets located on the island of Tenerife (Canary Islands, Spain) (Table 1). Likewise, to study the possible migration of macroelements from the heating of the container in the microwave, a total of 138 samples were taken and prepared according to the manufacturer’s indications.

### 2.2. Treatment of the Samples

A quantity of 10 g of sample, previously homogenized using a hand blender (Moulinex Powelix Life DD953D, Moulinex, Alençon, France), was obtained in porcelain capsules (Staatlich, Werheim, Germany).

The samples were dried in an oven (Nabertherm, Lilienthal, Germany) at a temperature of 80 °C for 24 h. The dry digestion process was carried out in a muffle oven (Nabertherm, Lilienthal, Germany) with the following temperature program: 420 °C, 48 h, temperature rise 50 °C/h, and maintenance of the temperature ramp for 24 h [11,12]. Ashes were obtained and dissolved in 1.5% HNO_3_ solution up to a final volume of 25 mL.

### 2.3. Analytical Determination and Quality Control

The macrolement determination was performed using ICP-OES (Inductively Coupled Plasma—Optical Emission Spectrometry) model ICAP 6300 Duo Thermo Scientific (Waltham, MA, USA), with the CETAX autosampler model ASX-520 (Thermo Fisher Scientific, Waltham, MA, USA) equipped with a CID86 chip (Charge Injection Device, Thermo Fisher Scientific, Waltham, MA, USA) (Table 2).

The quality control of the method was based on subjecting certain reference materials to the same conditions as the treatment of the samples [13]. The reference materials used were as follows: SRM 1515 apple leaves, SRM 1548a typical diet, and SRM 1567a wheat flour from the National Institute of Standards and Technology (NIST, Gaithersburg, MD, USA). The percentages of recovery of the analyzed elements were calculated, being in all cases greater than 98%.

The criteria, from an analytical point of view, that guaranteed the correct evaluation of the results presented in the work were the validation and the parameter of accuracy, which included the trueness of the method (established as recovery) and the precision of reproducibility (established as the coefficient of variation) applied to the reference materials SRM 1515 apple leaves, SRM 1548a typical diet, and SRM 1567a wheat flour. The required trueness (recovery) value was set at a minimum of 90%, and the reproducibility accuracy (coefficient of variation) was set at a maximum of 10%, with all metals under study meeting the required values. In addition, the specificity was also studied (which was carried out to check that the method was free of spectral interferences for each of the metals studied).

To establish the calibration linearity, the use of the relative calibration errors was used, setting as the acceptance criteria a maximum of 15% of this error for all metals in the lowest standards of each line and 10% in the rest of the points of the calibration lines.

The instrumental limits of detection and quantification were estimated based on the instrumental response. Specifically, they were determined by the analysis of 15 targets under reproducibility conditions.

### 2.4. Dietary Intake Assessment

To evaluate the dietary intake of the elements studied, the estimated daily intake (EDI) was calculated using Equation (1), the estimated daily intake calculation equation:

(1) $$EDI=Element\;concentration\;\left(\frac{mg}{kg}\right)\times Mean\;consumption\;(\frac{kg}{day})$$

After that, the percentage of contribution to the recommended daily intakes (RDI) was calculated (Equation (2), the percentage of contribution to the RDI equation):

(2) $$\%\;Contribution=\frac{EDI(\frac{mg}{day})}{Reference\;value}\times 100$$

The reference values used for the calculation of the different contribution percentages in the nutritional assessments were obtained from the EFSA reports (Table 3) [14].

### 2.5. Statistical Analysis

Statistical analysis was performed using GraphPad Prism 10.3.0 (GraphPad Software, Inc., Boston, MA, USA).

The results obtained were analyzed by the Shapiro–Wilk test, and the homogeneity of the variance was analyzed with the Levene test. An ANOVA analysis was run for data that followed a normal distribution, followed by a Tukey test. On the other hand, for data that did not present a normal distribution, a non-parametric Kruskal–Wallis and Mann–Whitney test was performed.

The study was carried out to determine the possible differences between the analyzed types of ready meals (animal, vegetable, and mixed origin) and between cooked vs. uncooked. Significant differences were considered values of *p* < 0.05.

## 3. Results and Discussion

### 3.1. Concentrations of Macroelements in Unheated and Microwaved Foods

In foods of animal origin, the highest concentrations of Na and Ca were recorded in the tails of garlic prawns (9381 ± 3102 mg Na/kg w.w. and 845 ± 134 mg Ca/kg w.w.). In the cases of K and Mg, both stood out for their high concentrations in the Spanish tortilla (omelette) (4432 ± 1260 mg K/kg w.w. and 520 ± 24.0 mg Mg/kg w.w.) (Table 4).

A pattern was observed in which all analyzed animal origin samples presented a high sodium concentration, especially prawns. This suggested that animal-based foods, particularly seafood, could contribute significantly to daily intake of Na. This requires special attention in order to avoid exceeding the recommendations for safe intake of Na.

A notable variation was also noted in potassium, magnesium, and calcium concentrations between different foods of animal origin, therefore indicating that a varied diet is essential to ensure a balanced intake of these minerals.

Meanwhile, in plant origin foods, Na and Mg stood out considerably in pesto pasta (4809 ± 1530 mg Na/kg w.w. and 644 ± 152 mg Mg/kg w.w.). The vegetable side dish stood out for its higher average concentration of K (3424 ± 1319 mg/kg fw). On the other hand, the average concentration of Ca recorded in chickpeas with spinach stood out, whose value was 742 ± 80.9 mg/kg w.w (Table 4).

It was observed that foods of plant origin presented a wide variability in the concentrations of macroelements, influenced by factors such as the type of vegetables and the other ingredients used. For example, preparations that included sauces or dressings may have had a higher sodium and fat content. In addition, legumes and leafy greens tended to be good sources of calcium and magnesium.

In mixed foods, pizza was the one with the highest average concentration of the four macroelements. The reasons may be due to the presence of various ingredients rich in these elements such as cheese, tomato sauce (which may contain concentrated tomatoes), and sausages, which are usually rich in salts and may contain additives; pizza dough may contain added salt, too.

The high concentration may be related to preservatives used in processed ingredients. The variability in macroelement content between different mixed-origin dishes suggested that the ingredients and the specific recipe of each microwave ready-to-eat meal played a crucial role in its nutritional profile, as might be expected. These patterns reflect the importance of considering ingredient composition and preparation methods when evaluating the macroelement content in microwave ready meals. Furthermore, it should be noted that significant differences (*p* < 0.05) were obtained between the three analyzed types of food (animal, vegetable, and mixed).

The following table shows the comparison between the average concentrations of uncooked food and once it was heated in the microwave as indicated by the manufacturer (Table 5). The analysis of macroelement concentrations in animal, plant, and mixed foods before and after microwave cooking revealed interesting patterns and some significant differences.

In foods of animal origin, in the case of Na, a general increase in concentrations was observed after cooking, being more noticeable in meatballs (from 5723 to 7204 mg/kg fw) and shredded meat (from 5110 to 5589 mg/kg fw). Meanwhile, K showed mixed variations, with increases in some foods such as meatballs and shredded meat but decreases in others such as omelettes and prawns. On the other hand, Mg concentrations remained relatively stable, with minor changes. As for Ca, increases were observed in most foods, except for chicken curry and prawns. It should also be noted that significant differences were recorded in the case of Ca content in most foods of animal origin (*p* < 0.05).

In the case of Na in plant-based foods, there was a significant increase in the vegetable garnish, round rice, and vegetable cream soup but a notable decrease in chickpeas with spinach. As for K, it showed increases in most foods, except for chickpeas with spinach. For Mg, it presented significant increases in cream of vegetable soup and round rice but a notable decrease in chickpeas with spinach. And in the case of Ca, variable changes were observed, with a significant increase in cream of vegetables but a decrease in chickpeas with spinach. It should be noted that significant differences were recorded in the case of Ca content in most plant-based foods (*p* < 0.05).

Finally, in the group of foods of mixed origin, a general increase in Na concentrations was observed after cooking, being more noticeable in pizza and cannelloni. K increases were shown in most foods, except cannelloni. As for Mg, it displayed increases in most foods, except for cannelloni. Significant increases in Ca were observed in pizza and seafood paella, but a notable decrease was observed in cannelloni. It should be noted that significant differences were recorded in the case of Ca content in most mixed foods (*p* < 0.05).

These differences in macroelement concentrations between raw and cooked samples suggested that the microwave cooking process could significantly affect the nutritional content of food. The observed changes could be attributed to possible interactions with the containers used in the microwave [15,16,17,18,19,20]. It is important to note that pesto pasta in plant-based foods could not be tested after cooking since it was withdrawn from the market, which limited the complete comparison in this group.

### 3.2. Comparison with Other Authors

Table 6 shows the concentrations of the analyzed macroelements obtained by other authors and their comparison with the results obtained in the present study.

Regarding the macroelement content of foods of animal origin, higher concentrations of Na, Ca, and Mg were registered in the present study (2025) compared with the other consulted bibliography. However, the mean K concentrations in chicken and meatballs were lower than the ones found in other studies.

With respect to the macroelement content of foods of plant origin, higher concentrations of Ca and Mg were observed in the data provided by the present analysis than in the data of the other authors in all foods. However, the mean concentrations of Na were higher in our analysis in pasta and rice and lower in cream of prepared vegetables, having a value of almost half of the average concentration of other authors. The same went for K, with our concentration value being much higher for rice but lower for cream of vegetable soup.

Regarding the average concentrations of macroelements in foods of mixed origin, higher concentrations of the four macroelements were observed in the data analyzed in the present study compared with other authors, highlighting the concentrations of Na analyzed in our samples, which had much higher values for the same food.

### 3.3. Exposure Assessment

The exposure assessment was based on calculation of the EDI (Equation (1)). In order to do so, data on the average daily consumption of ready meals provided by AESAN [33] and the ENALIA 2 project (National Survey of Food) in the adult, older adult, and pregnant population were used [34]. Table 7 shows the EDI values for each macroelement according to the average consumption of the analyzed dish (Table 7).

### 3.4. Nutricional Assessment

Once the EDI values were obtained, the reference values established by the EFSA (European Food Safety Authority) in its various reports were used. These recommended exposures could be found in its online application “DRV Finder”, including the adequate intake (AI) for Mg, K, and Na and the average requirement (AR) for Ca as there is not a established AI for this element by EFSA [14].

Table 8 indicates the percentages of contribution to the average recommended intake (AR) for Ca. The percentages obtained were, in general, quite low for most of the analyzed foods. Only pizza managed to contribute more than 22% for children and more than 25% for adults. These percentages were considered significant since they represented a percentage of a quarter of the Ca that should be consumed daily through the diet.

Table 9 shows the contribution percentages for the Mg. In this case, based on the AI (adequate intake) established by the EFSA, omelettes were the biggest dietary source of Mg among the studied foods (22.3–31.2%); however, this was not the only significant dietary source of Mg as pizza ranged between 12.5% and 14.6% of the Mg AI, similar to the analyzed vegetable garnish (19.3–27%). 

Table 10 indicates the percentages of contribution to adequate potassium intake. According to the obtained percentages, it was found that the omelette offered the highest contributions to the potassium AI, for all age groups and sexes. Thus, in the case of children aged 11 to 17 years, a percentage higher than 24% was obtained, while for adults it was 19.0%. This contribution could be considered significant in terms of K intake.

Finally, Table 11 shows the percentages of contribution to adequate and safe intake of Na, which was established at 2 g/day for children older than 11 years of age and adults. It should be noted that in this case, the AI referred to a safe intake, beyond which there could be a risk to the health of consumers.

Looking at the percentages of contribution obtained, the consumption of tortillas represented a very high contribution, close to 50% of the total Na that was expected to be consumed daily. Likewise, for the contribution from pizza, close to 40% of the AI, consumption was also noteworthy. It is necessary to remember that Na is one of the elements that can pose the most risks to cardiovascular health as it is related to hypertension, and low consumption is recommended in cases of certain pathologies.

Therefore, the intake of the analyzed omelette (48.9%), pizza (38.7%), meatballs (26.1%), chicken curry (25.1%), cream of vegetables (20.7%), and vegetable garnish (18.5%) in the daily quantity estimated may pose a risk of Na dietary overexposure, and therefore, further research would be of interest as the percentage of contribution to the safety exposure larger than 20% was reached with just one food type.

### 3.5. Recommendations for the Consumer Population of Microwave Ready-to-Eat Meals

According to the data presented in the present study, it is recommended that the population should not abuse these products, since the percentages of contribution to some of the macroelements analyzed such as Na indicated high values. That is why it is recommended to avoid certain ready-to-eat dishes prepared by those who suffer from cardiovascular diseases such as hypertension [35,36,37,38,39].

The population is urged to review the labeling of foods prepared for microwaves since in some cases the presence of certain ingredients or additives can increase the intake of Na.

As for the rest of the macroelements, the contributions from the consumption of the analyzed dishes may present in some cases a significant contribution to the recommended or adequate intakes, being positive for the health of consumers; however, it is always preferable to eat fresh food prepared with culinary techniques that maintain the nutritional value of the food.

Likewise, given the notable differences between dishes of animal, vegetable, and mixed origin, the consumer population is reminded that it is necessary to vary the diet and not depend monotonously on a single type of dish.

## 4. Study Limitations and Future Developments

The main limitation of the present study is the fact that the studied food may not be representative of global gastronomy, as it focuses on dishes prepared and marketed in Spain. Therefore, a specific legislation is applied that may only be also applied to the other European countries, even though their consumption patterns may differ.

For this reason, one of the future research projects in relation to these products should not only be the study and analysis of a greater variety of elements but also trying to achieve data from dishes that are representative of a more global gastronomy.

## 5. Conclusions

The obtained results showed that sodium was the macroelement with the highest presence in all the analyzed foods, with the omelette (48.9% of the Na AI in a consumption of 150 g/day) followed by pizza with a contribution of 38.7% to the reference value of Na being the more noteworthy dishes.

Potassium had its highest contributions in foods such as pre-cooked vegetables (12.2%) and omelettes (17.5%), while rice registered the lowest values (0.87%). Calcium reached its maximum values in pizza (22.7%) and chickpeas with spinach (742 mg/kg), with minimums in seafood paella (1.48%).

Meanwhile, magnesium showed its highest contribution in pre-cooked vegetables (22.5% in males and 19.8% in females) and pizza (14.6% in males and 12.51% in females), while the lowest values corresponded to basmati rice (4.85% in males and 4.15% in females).

In general, microwave ready meals showed a high Na concentration, which could lead to a risk of hypertension if not consumed with moderation, while the variations observed in the macroelements among the analyzed food highlight the importance of a balanced and diversified diet. Therefore, this analysis provides significant data to evaluate the nutritional quality of these foods, considering their increasing consumption in recent years and their possible risk.

## Figures and Tables

**Table 1:** Samples of analyzed microwave ready-to-eat dishes without cooking.

Samples of Animal Origin		Plant-Based Samples		Mixed Samples	
Omelette (eggs)	10	Garnish of roasted vegetables (vegetables and vegetables)	10	Pizza	10
Chicken curry (fowl)	10	Round rice (cereals)	10	Lasagna	10
Prepared meatballs (pork)	10	Pesto pasta (derived from cereals)	10	Mixed paella	10
Shredded meat (veal)	10	Cream of vegetables (vegetables)	10	Cannelloni	10
Prawn tails (crustacean)	10	Chickpeas with spinach (legumes)	10	Spaghetti Bolognese	10
Total no.	50	Total no.	50	Total no.	50

**Table 2:** Instrumental conditions of the ICP-OES equipment.

Element	Wavelength (nm)	LOQ (mg/L)	LOD (mg/L)
Ca	315.887	5.432	1.629
Mg	383.826	5.268	1.58
K	766.490	5.883	1.764
Na	818.326	7.404	2.221

**Equipment instrumental conditions:** RF power (1.2 KW), gas flow (0.5 L/min), pump injection speed (50 rpm), and stabilization time (0 s). Carrier gas: high-purity liquid argon (99.999%) (Air Liquid, Madrid, Spain).

**Table 3:** Dietary references values established by EFSA for the studied elements.

**Calcium**	**Target Population**	**Age (Years)**	**Sex**	**AR (mg/Day)**
	Children	10–17	Both	960
	Adults	18–24		860
		≥25		750
**Magnesium**	**Target Population**	**Age (Years)**	**Sex**	**AI (mg/Day)**
	Children	10–17	Male	300
			Female	250
	Adults	≥18	Male	350
			Female	300
**Potassium**	**Target Population**	**Age (Years)**	**Sex**	**AI (mg/Day)**
	Children	11–14	Both	2700
	Children	15–17		3500
	Adults	≥18		3500
**Sodium**	**Target Population**	**Age (Years)**	**Sex**	**Safe and AI (g/Day)**
	Children	11–17 years	Both	2
	Adults	≥18 years		

**Table 4:** Macroelement mean concentration (mg/kg w.w.) in the unheated analyzed samples.

Animal Origin	Na	K	Mg	Ca
Omelette	6522 ± 1441	4432 ± 1260	520 ± 24.0	373 ± 28.8
Chicken curry	5498 ± 1384	1730 ± 572	307 ± 40.3	335 ± 142
Meatballs	5723 ± 1036	2214 ± 733	426 ± 24.9	368 ± 27.9
Shredded meat	5110 ± 653	2690 ± 437	389 ± 40.7	199 ± 43.2
Prawns	9381 ± 3102	750 ± 275	370 ± 40.6	845 ± 134
**Plant Origin**				
Vegetable garnish	2971 ± 967	3424 ± 1319	541 ± 69.9	327 ± 27.9
Round rice	1762 ± 464	526 ± 231	251 ± 45.6	95.0 ± 12.2
Pesto pasta	4809 ± 1530	1475 ± 424	644 ± 152	365 ± 41.1
Cream of vegetables	2589 ± 656	2028 ± 520	181 ± 15.6	269 ± 23.3
Chickpeas with spinach	4754 ± 968	2596 ± 487	530 ± 50.4	742 ± 80.9
**Mixed Origin**				
Pizza	8856 ± 1873	2816 ± 723	501 ± 35.4	2468 ± 477
Lasagna	4586 ± 831	2335 ± 598	369 ± 34.8	1791 ± 348
Seafood paella	4842 ± 1518	1280 ± 352	396 ± 328	242 ± 85.3
Cannelloni	4344 ± 758	1788 ± 477	386 ± 23.9	938 ± 98.1
Spaghetti Bolognese	4087 ± 1036	2227 ± 835	399 ± 43.1	324 ± 18.5

**Table 5:** Comparison of the concentration of macroelements before and after the microwave process.

Food	Na (mg/kg w.w.)		K (mg/kg w.w.)		Mg (mg/kg w.w.)		Ca (mg/kg w.w.)	
	Before	After	Before	After	Before	After	Before	After
**Animal Origin**								
Omelette	6522	6560 ± 1783	4432	4084 ± 1141	520	523 ± 46.5	373	416 ± 40.3
Chicken curry	5498	5893 ± 1244	1730	1818 ± 448	307	285 ± 20.0	335	273 ± 19.8
Meatballs	5723	7204 ± 1160	2214	2944 ± 426	426	438 ± 51.1	368	389 ± 46.3
Shredded meat	5110	5589 ± 1188	2690	3204 ± 944	389	429 ± 70.7	199	242 ± 35.5
Prawns	9381	9989 ± 2631	750	655 ± 891	370	352 ± 38.2	844	680 ± 157
**Plant Origin**								
Vegetable garnish	2971	3868 ± 1327	3424	3906 ± 903	541	550 ± 126	327	314 ± 90.7
Round rice	1762	2143 ± 231	526	687 ± 90.9	251	298 ± 31.1	95	152 ± 12.3
Cream of vegetables	2588	3800 ± 277	2028	2138 ± 175	181	511 ± 42.2	269	760 ± 128
Chickpeas with spinach	4754	2734 ± 1295	2596	2070 ± 913	530	220 ± 78.2	742	326 ± 107
**Mixed Origin**								
Pizza	8858	10870 ± 676	2816	3031 ± 150	501	591 ± 35.6	2468	3235 ± 175
Lasagna	4586	5293 ± 885	2335	2972 ± 471	369	449 ± 82.5	1791	2045 ± 311
Seafood paella	4842	4498 ± 877	1280	2023 ± 587	396	424 ± 60.8	242	931 ± 163
Cannelloni	4344	6401 ± 2378	1788	1376 ± 540	386	353 ± 260	938	285 ± 120
Spaghetti Bolognese	4087	4862 ± 1432	2227	2372 ± 682	399	443 ± 98.3	324	349 ± 107

**Table 6:** Macroelement concentrations (mg/kg) found by different authors and comparison with the obtained data.

*Food*	*Na*	*K*	*Ca*	*Mg*	*References*
*Omelette*	2788	1402	315	120	[21,22]
	6521	4432	373	520	This study, 2025
*Chicken*	515.5	1809	41.2	191.9	[23,24,25]
	5498	1731	335	307	This study, 2025
*Meatballs*	549	3753	54.1	202.7	[23,26]
	5723	2214	368	426	This study, 2025
*Pesto pasta*	241	1467	305	126	[27]
	4809	1476	365	644	This study, 2024
*Rice*	2	250	47	95	[28,29,30]
	1762	526	95.0	251	This study, 2025
*Cream of vegetables*	4360	2730	162	137	[26,30]
	2589	2028	269	181	This study, 2025
*Pizza*	818	1750	2190	470	[27,31]
	8858	2816	2568	501	This study, 2025
*Lasagna*	3798	2015	845	185	[27,32]
	4586	2335	1791	369	This study, 2025

**Table 7:** EDI (mg/day) values for the analyzed samples according to the mean consumption.

	EDI (mg/day)			
**Animal**	**Na**	**K**	**Mg**	**Ca**
Omelette	978	665	78.0	56.0
Chicken curry	502	158	28.0	30.6
Meatballs	522	202	38.9	33.6
Shredded meat	272	143	20.7	10.6
Prawns	114	9.13	4.50	10.3
**Plant Origin**	**Na**	**K**	**Mg**	**Ca**
Vegetable garnish	371	427	67.5	40.8
Round rice	102	30.5	14.6	5.51
Pesto pasta	257	78.9	34.4	19.5
Cream of vegetables	413	324	28.9	43.0
Chickpeas with spinach	217	119	24.2	33.9
**Mixed Origin**	**Na**	**K**	**Mg**	**Ca**
Pizza	774	246	43.8	216
Lasagna	244	124	19.6	95.4
Seafood paella	281	74.3	23.0	14.0
Cannelloni	231	95.2	20.6	49.9
Spaghetti Bolognese	219	119	21.3	17.3

Average consumption data (g/day): omelette (150), chicken curry (91.26), meatballs (91.26), shredded meat (53.25), prawns (12.17), rice (58.02), pesto pasta (53.49), cream of vegetables (159.68), chickpeas with spinach (45.68), pizza (87.42), lasagna (53.25), seafood paella (58.02), cannelloni (53.25), and spaghetti Bolognese (53.49) [33,34].

**Table 8:** Contribution percentages (%) to the calcium AR.

	Animal Origin	Children (10–17 Years)	Adults (18–24 Years)	Adults (≥25 Years)
**Contribution Percentage (%)**	Omelette	5.77	6.51	7.37
	Chicken curry	3.16	3.55	4.03
	Meatballs	3.47	3.91	4.42
	Shredded meat	1.09	1.23	1.40
	Prawns	1.06	1.20	1.35
	**Plant Origin**			
	Vegetable garnish	4.21	4.75	5.38
	Round rice	0.57	0.64	0.73
	Pesto pasta	2.01	2.27	2.57
	Cream of vegetables	4.43	4.99	5.66
	Chickpeas with spinach	3.50	3.94	4.47
	**Mixed Origin**			
	Pizza	22.2	25.1	28.4
	Lasagna	9.84	11.1	12.6
	Seafood paella	1.45	1.63	1.85
	Cannelloni	5.15	5.81	6.58
	Spaghetti Bolognese	1.79	2.02	2.28

**Table 9:** Contribution percentages (%) to the magnesium AI.

		Children (11–17 Years)		Adults (≥18 Years)	
**Contribution Percentage (%)**	**Animal Origin**	**Male**	**Female**	**Male**	**Female**
	Omelette	26.0	31.2	22.3	26.0
	Chicken curry	9.34	11.2	8.00	9.34
	Meatballs	13.0	15.6	11.1	13.0
	Shredded meat	6.90	8.29	5.92	6.90
	Prawns	1.50	1.80	1.29	1.50
	**Plant Origin**				
	Vegetable garnish	22.5	27.0	19.3	22.5
	Round rice	4.85	5.83	4.16	4.85
	Pesto pasta	11.5	13.8	9.84	11.5
	Cream of vegetables	9.63	11.6	8.26	9.63
	Chickpeas with spinach	8.07	9.68	6.92	8.07
	**Mixed Origin**				
	Pizza	14.6	17.5	12.5	14.6
	Lasagna	6.55	7.86	5.61	6.55
	Seafood paella	7.66	9.19	6.56	7.66
	Cannelloni	6.85	8.22	5.87	6.85
	Spaghetti Bolognese	7.11	8.54	6.10	7.11

**Table 10:** Contribution percentages (%) to the potassium AI.

	Lactating Females	≥18	Female	4000	
**Contribution Percentage (%)**	**Animal Origin**	**Children** **(11–17 Years)**	**Children** **(15–17 Years)**	**Adults** **(≥18 Years)**	**Lactating Females** **(≥18 years)**
	Omelette	24.6	19.0	19.0	16.6
	Chicken curry	5.85	4.51	4.51	3.95
	Meatballs	7.48	5.77	5.77	5.05
	Shredded meat	5.31	4.09	4.09	3.58
	Prawns	0.34	0.26	0.26	0.228
	**Plant Origin**				
	Vegetable garnish	15.8	12.2	12.2	10.7
	Round rice	1.13	0.87	0.87	0.763
	Pesto pasta	2.92	2.25	2.25	1.97
	Cream of vegetables	11.99	9.25	9.25	8.10
	Chickpeas with spinach	4.39	3.39	3.39	2.96
	**Mixed Origin**				
	Pizza	9.12	7.03	7.03	6.15
	Lasagna	4.61	3.55	3.55	3.11
	Seafood paella	2.75	2.12	2.12	1.86
	Cannelloni	3.53	2.72	2.72	2.38
	Spaghetti Bolognese	4.41	3.40	3.40	2.98

**Table 11:** Contribution percentages (%) to the sodium safe intake and AI.

	Animal Origin	Children (11–17 Years)	Adults (18–24 Years)
**Contribution Percentage (%)**	Omelette	48.9	48.9
	Chicken curry	25.1	25.1
	Meatballs	26.1	26.1
	Shredded meat	13.6	13.6
	Prawns	5.7	5.7
	**Plant Origin**		
	Vegetable garnish	18.5	18.5
	Round rice	5.1	5.1
	Pesto pasta	12.9	12.9
	Cream of vegetables	20.7	20.7
	Chickpeas with spinach	10.9	10.9
	**Mixed Origin**		
	Pizza	38.7	38.7
	Lasagna	12.2	12.2
	Seafood paella	14.0	14.0
	Cannelloni	11.6	11.6
	Spaghetti Bolognese	10.9	10.9

## Data Availability

The original contributions presented in the study are included in the article; further inquiries can be directed to the corresponding author.
